# Combined *in vitro* effects of temozolomide and erucic acid on U87 MG cell viability, oxidative stress, and expression of genes involved in apoptotic signalling

**DOI:** 10.2478/aiht-2026-77-4098

**Published:** 2026-06-30

**Authors:** İbrahim Gecili, Sıdıka Genç, Ahmet Hacımüftüoğlu, Adem Güner, Esmanur Niğde, Kübra Karabulut, Dündar Okan Yıllar, Ali Taghizadehehghalehjoughi, Abdelaty Mostafa Abdelaty Hassibelnaby

**Affiliations:** Istanbul Beykent University Faculty of Medicine, Department of Medical Pharmacology, Istanbul, Turkey; Bilecik Şeyh Edebali University Faculty of Medicine, Department of Medical Pharmacology, Bilecik, Turkey; Atatürk University Medical Faculty, Department of Medical Pharmacology, Erzurum, Turkey; Sinop University Faculty of Health Sciences, Department of Occupational Health and Safety, Sinop, Turkey; Cairo University Faculty of Veterinary Medicine, Department of Pharmacology, Giza, Egypt

**Keywords:** cancer therapy, chemotherapy, cytotoxicity, fatty acids, glioblastoma, oxidative stress, citotoksičnost, kemoterapija, masne kiseline, oksidacijski stres, terapija raka

## Abstract

Limited efficacy of glioblastoma treatment with temozolomide (TMZ) due to resistance has prompted researchers to look for adjuvants capable of enhancing TMZ-associated cytotoxicity. The aim of our *in vitro* study was to evaluate such effects of erucic acid when combined with TMZ in human U87 MG cells. Cells were treated for 24 or 72 h with vehicle (control), TMZ (200 ng/mL), or TMZ plus EA (20, 40, 80, or 160 µg/mL). Cell viability was assessed using the MTT assay, membrane integrity with lactate dehydrogenase (LDH) release, and intracellular redox balance by measuring total antioxidant capacity (TAC) and total oxidant status (TOS). The mRNA expression of apoptosis-related genes (*p53, p21, Bax, Bcl-2, Bcl-xL*, and *caspase-3*) was analysed by reverse transcription-quantitative polymerase chain reaction (RT-qPCR). Compared with the vehicle control, TMZ plus EA reduced cell viability and increased LDH release, with more pronounced effects at higher EA concentrations and at 72 h. These changes were accompanied by lower TAC and higher TOS, indicating a shift toward a pro-oxidant intracellular environment. At the transcriptional level, the combination induced increased pro-apoptotic Bax, caspase-3, p53, and p21 expression and reduced Bcl-2 and Bcl-xL expression. In conclusion, EA potentiated TMZ-associated cytotoxic responses in U87 MG cells with accompanying oxidative imbalance and apoptosis-related transcriptional changes.

## Abstract (hr)

Ograničena djelotvornost liječenja glioblastoma temozolomidom (TMZ) zbog nastanka rezistencije potaknula je potragu za adjuvansima koji će pojačati njegovu citotoksičnost. Cilj našeg *in vitro* istraživanja bio je na humanim stanicama glioblastoma U-87 MG procijeniti ima li eruka kiselina takav učinak u kombinaciji s TMZ-om. Stanice smo izložili DMSO (kontrola), TMZ-u (200 ng/mL) ili kombinaciji TMZ-a i eruka kiseline (20, 40, 80 ili 160 µg/mL) 24 sata odnosno 72 sata. Vijabilnost stanica utvrđena je MTT testom, a integritet membrane, otpuštanje laktat-dehidrogenaze (LDH) i unutarstanična redoks ravnoteža procijenjeni su mjerenjem ukupnog antioksidacijskog kapaciteta (engl. *total antioxidant capacity*; TAC) i ukupnog oksidacijskog statusa (engl. *total oxidant status*; TOS). Ekspresija mRNA gena povezanih s apoptozom (p53, p21, Bax, Bcl-2, Bcl-xL i kaspaza-3) analizirana je metodom kvantitativne lančane reakcije polimerazom nakon reverzne transkripcije (RT-qPCR). U usporedbi s kontrolom, kombinacija TMZ-a i eruka kiseline smanjila je vijabilnost stanica i povećala otpuštanje LDH-a, pri čemu su učinci bili izraženiji pri višim koncentracijama eruka kiseline i nakon 72 sata. Ove promjene popraćene su nižim TAC-om i višim TOS-om, što upućuje na pomak prema prooksidacijskom unutarstaničnom okolišu. Na transkripcijskoj razini kombinacija je inducirala povećanu ekspresiju proapoptotskih proteina Bax, kaspaza-3, p53 i p21 te smanjenu ekspresiju Bcl-2 i Bcl-xL. Naši rezultati potvrđuju da eruka kiselina pojačava citotoksičnost TMZ-a za stanice U-87 MG kroz oksidacijski stres, što objašnjava indukciju apoptotskih signalnih putova.

Glioblastoma is the most common malignant primary brain tumour in adults, and despite the current standard treatment consisting of surgical resection, radiotherapy, and temozolomide (TMZ) regimen, median survival remains limited to approximately 14–16 months (1). This prognosis underscores the need to explore adjuvants that can potentiate the cytotoxic effects of TMZ and modulate resistance pathways rather than completely replace TMZ (2).

One promising candidate is erucic acid (EA, *cis*-13-docosenoic acid), a long-chain omega-9 fatty acid found in rapeseed and related oil-bearing seeds. Recent experimental studies focused on EA in the context of cancer biology have demonstrated that it can suppress proliferation, increase oxidative stress, and potentiate cell death responses consistent with apoptosis and ferroptosis in several solid tumour models. When used in combination with anticancer agents, EA has been shown to increase their cytotoxicity (3,4,5,6,7). However, to the best of our knowledge, little is known about the effects of EA in combination with TMZ in U87 MG cells, as the existing work on lipid-based strategies has more often focused on fatty acids or TMZ-fatty acid conjugates other than EA (8).

This study aimed to address this gap by determining the effects of combined TMZ and EA treatment on glioblastoma cell viability, membrane damage (LDH), oxidative balance (TAC/TOS), and the expression of apoptotic genes (*p53*, *p21*, *Bax*, *Bcl-2*, *Bcl-xL*, and *caspase-3*). We hypothesised that combined TMZ and EA treatment would intensify cytotoxic and oxidative stress-related responses and promote a more pro-apoptotic gene expression profile under the present experimental conditions.

## MATERIALS AND METHODS

This *in vitro* study was conducted at the Bilecik Şeyh Edebali University Faculty of Medicine, Department of Medical Pharmacology in accordance with institutional guidelines for cell culture research.

### Chemicals

All the chemicals used in the study were of analytical grade. Cell culture reagents included Dulbecco’s Modified Eagle Medium (DMEM; Cat. No. 31330038, Thermo Fisher Scientific, Waltham, MA, USA), foetal bovine serum (FBS; Cat. No. 10500064, Thermo Fisher Scientific), and penicillin-streptomycin (Cat. No. 15140122, Thermo Fisher Scientific).

TMZ (≥98 % purity; CAS No. 85622-93-1) and EA (~90 % purity; CAS No. 112-86-7) were obtained from Sigma-Aldrich (Burlington, MA, USA).

Additional reagents included the reverse transcription-quantitative polymerase chain reaction (RT-qPCR) primers obtained from EcoTech Biotechnology (Erzurum, Turkey; Cat. No. ERT200), RNA stabilisation solution (EcoTech Biotechnology; Cat. No. E2075), SYBR Master Mix (EcoTech Biotechnology; Cat. No. SMM5), 3-(4,5-dimethylthiazol-2-yl)-2,5-diphenyltetrazolium bromide (MTT; Sigma-Aldrich, Cat. No. M5655), B27 supplement (Thermo Fisher Scientific; Cat. No. 17504044), phosphate-buffered saline (PBS; Thermo Fisher Scientific, Cat. No. 70011044), total antioxidant capacity (TAC) assay kit (Rel Assay Diagnostics, Şehitkamil/Gaziantep, Turkey; Cat. No. RL0017), total oxidant status (TOS) assay kit (Rel Assay Diagnostics; Cat. No. RL0024), and human lactate dehydrogenase A (LDHA) ELISA kit (Feiyue Biotech, Wuhan, China; Cat. No. FY-EH1311S).

All the solvents, including dimethyl sulphoxide (DMSO; Thermo Fisher Scientific, Cat. No. 20688), were freshly aliquoted and stored under argon to minimise oxidative degradation.

### Glioblastoma cell culture and experimental groups

The U87 MG cell line used in this study was obtained from the American Type Culture Collection (ATCC; Manassas, VA, USA; Cat. No. HTB-14). The cells were cultured in DMEM supplemented with 10 % FBS and 1 % penicillin-streptomycin and maintained at 37 °C in a humidified atmosphere containing 5 % CO_2_.

TMZ and EA stock solutions were prepared in DMSO and diluted in complete DMEM immediately before use. The final DMSO concentration was kept constant at 0.01 % (v/v) in all the wells.

The control group received vehicle only [complete DMEM containing 0.01 % (v/v) DMSO], whereas treatment groups received TMZ (200 ng/mL) alone or in combination with EA (20, 40, 80, or 160 µg/mL), hereafter referred to as TMZ+EA20, TMZ+EA40, TMZ+EA80, and TMZ+EA160.

Rather than employing an additional exogenous positive control, TMZ monotherapy was used as the internal reference benchmark for cytotoxicity- and oxidative stress-related outcomes. The selected TMZ concentration of 200 ng/mL was based on preliminary concentration-response experiments and prior literature demonstrating reproducible cytotoxic effects in U87 MG cells (9, 10)

This study was designed to determine whether EA enhances the magnitude or profile of TMZ-induced cellular responses under standardised *in vitro* conditions rather than to validate specific cell death pathways. The EA concentration range from 20 to 160 µg/mL (approximately 59–473 µmol/L) used in this study was based on concentration-related cytotoxicity reported by Sharma et al. in N2a neuroblastoma cells (11).

In all the assays, the cells were exposed to TMZ or TMZ+EA combinations for 24 h or 72 h, and all the biochemical and molecular analyses were performed at both time points. The 24 h time point was selected to evaluate the early cellular effects and the 72 h time point delayed apoptotic response due to accumulated DNA damage during cell division.

### MTT assay

Cell viability was assessed using the MTT assay following the procedure reported by Mosmann (12). U87 MG cells were seeded into 96-well plates and treated as described above. At each time point (24 h and 72 h), the culture medium was removed and replaced with 10 µL of MTT reagent plus 90 µL of complete culture medium per well. The plates were incubated for 4 h to allow for formazan formation. Formazan crystals were then dissolved by adding 100 µL of DMSO to each well, and the absorbance was measured at 570 nm using a SpectraMax i3x microplate reader (Molecular Devices, San Jose, CA, USA).

### LDH-ELISA

The enzyme-linked immunosorbent assay (ELISA) was used to measure human LDH release as an indicator of cell membrane damage. After treatment, we collected culture supernatants and centrifuged them at 12,000 *g* and 4 °C for 10–15 min to remove cellular debris. LDH levels were quantified according to the manufacturer’s instructions, and the absorbance was measured at 450 nm using the SpectraMax i3x microplate reader (Molecular Devices). The results are expressed in percentages relative to the vehicle control group.

### Total antioxidant capacity

Total antioxidant capacity (TAC) was measured in cell lysate supernatants using the above-mentioned commercial kit (Rel Assay Diagnostics), based on Erel’s colorimetric method (13). In this assay, antioxidants in the sample decolourise the 2,2′-azino-bis(3-ethylbenzothiazoline-6-sulphonic acid radical cation (ABTS^•+^), and the change in absorbance is measured at 660 nm. TAC values were calculated according to the manufacturer’s instructions, expressed as mmol/L Trolox equivalents, and reported as percentages relative to the vehicle control group.

### Total oxidant status

Total oxidant status (TOS) was also measured in cell lysate supernatants using the above-mentioned commercial kit (Rel Assay Diagnostics), based on Erel’s colorimetric method (14). Oxidant species in the sample oxidise ferrous ions to ferric ions under acidic conditions, and the ferric ions form a coloured complex with xylenol orange. The absorbance was measured at 530 nm. The assay was calibrated with hydrogen peroxide standards, and the results are expressed as µmol of H_2_O_2_ equivalents per litre and reported as percentages relative to the vehicle control group.

### Real-time qPCR analysis

RNA was isolated from U87 MG cells using the EcoPURE Total RNA Kit (EcoTech Biotechnology). The quality and concentration of the extracted RNA were verified before complementary DNA (cDNA) synthesis using reverse transcription to obtain cDNA templates suitable for quantitative gene expression analysis.

The mRNA expression of p53, p21, Bax, Bcl-2, Bcl-xL, and caspase-3 was quantified using the SYBR Green-based RT qPCR with SYBR Master Mix. For internal reference we used glyceraldehyde 3-phosphate dehydrogenase (GAPDH), as its expression remained stable across the experimental conditions. Amplification reactions were conducted under optimised thermal cycling conditions, and fluorescence signals were collected throughout the amplification process. For each sample, the ΔCt values were calculated as Ct(target) minus Ct(GAPDH). Relative gene expression levels were calculated using the 2^-ΔΔCt^ method, with the control group serving as the calibrator. For statistical analysis, we used ΔCt values to minimise potential distributional bias associated with the fold change.

### Statistical analyses

Data are presented as the means ± standard deviations (SD), unless stated otherwise. For each assay (MTT, LDH-ELISA, TAC, and TOS), *n* denotes the number of independent biological replicates (independent experiments performed on different days), and technical replicates within each experiment were averaged to yield one value per biological replicate. Group differences at each time point (24 h and 72 h) were analysed separately using one-way analysis of variance (ANOVA). When ANOVA indicated an overall group effect, we ran multiple comparisons vs control using Dunnett’s test (two-sided).

All analyses were run on GraphPad Prism version 9.5.1 (GraphPad Software, San Diego, CA, USA). P values <0.05 were considered statistically significant.

## RESULTS

### Cell viability, LDH levels, and morphology

**Figure 1:**
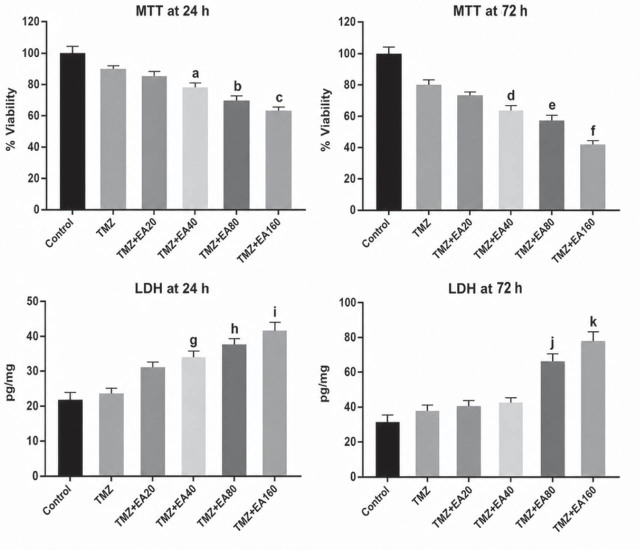
Cell viability and LDH release following the treatment of U87 MG cells with TMZ (200 ng/mL) alone or in combination with EA (20, 40, 80, and 160 µg/mL) for 24 h and 72 h. The data are presented as means ± SD of 8 independent biological replicates. Within each replicate, five technical wells were averaged to obtain one value. ^a^ P=0.028; ^b^ P=0.019; ^c^ P=0.006; ^d^ P=0.004; ^e^ P=0.002; ^f^ P=0.0009; ^g^ P=0.029; ^h^ P=0.018; ^i^ P=0.004; ^j^ P=0.006; ^k^ P=0.0008 compared to control (one-way ANOVA followed by Dunnett’s test). EA – erucic acid; TMZ – temozolomide

Figure 1 shows a significant drop in cell viability vs control in the first 24 h of combined TMZ+EA exposure, starting with the 40 µg/mL EA concentration and higher (80 µg/mL and 160 µg/mL). At 72 h, cell viability further decreased in these groups.

Conversely, LDH release increased significantly starting with the EA concentration of 40 µg/mL at 24 h but remained significant only for the 80 and 160 µg/mL concentrations at 72 h.

**Figure 2:**
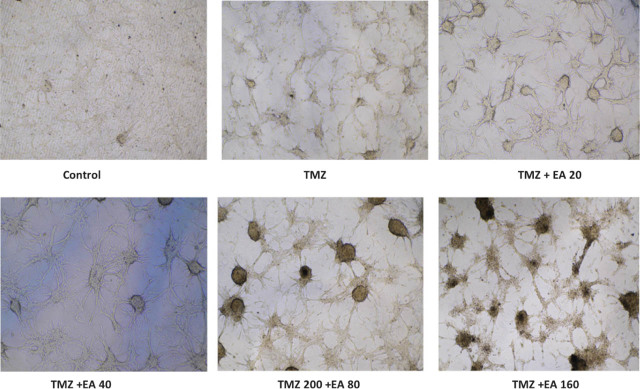
Representative phase-contrast images showing morphological changes in U87 MG cells following treatment with TMZ (200 ng/mL) alone or in combination with EA (20, 40, 80, and 160 µg/mL) for 72 h. Images were acquired using a Nikon ELWD 20× objective (N.A. 0.3) with a 10× ocular lens, yielding a total magnification of 200×. EA – erucic acid; TMZ – temozolomide

Figure 2 shows morphological changes in treated U87 MG cells after 72 h. Control cells displayed a widespread, cohesive morphology with fine cellular extensions, whereas those treated with TMZ alone showed reduced density and clear signs of cellular stress. The addition of increasing concentrations of EA to TMZ resulted in even more pronounced morphological deterioration, characterised by cell shrinkage, cytoplasmic condensation, and loss of intercellular connections. In the TMZ+EA80 and 160 groups, the cells became rounded, cellular extensions largely disappeared, and morphological features became compatible with apoptotic changes.

### Oxidative balance

**Figure 3:**
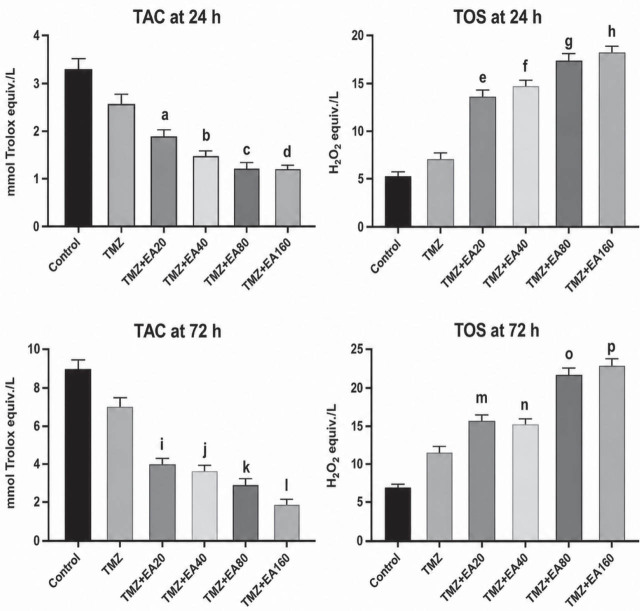
TAC and TOS levels following the treatment of U87 MG cells with TMZ (200 ng/mL) alone or in combination with EA (20, 40, 80, and 160 µg/mL) for 24 h and 72 h. The data are presented as means ± SD of 8 independent biological replicates. Within each replicate, five technical measurements were averaged to obtain one value. ^a^ P=0.041; ^b^ P=0.014; ^c^ P=0.009; ^d^ P=0.004; ^e^ P=0.047; ^f^ P=0.028; ^g^ P=0.009; ^h^ P=0.006; ^i^ P=0.006; ^j^ P=0.004; ^k^ P=0.002; ^l^ P=0.0007; ^m^ P=0.008; ^n^ P=0.006; ^o^ P=0.002; ^p^ P=0.0009 compared to control (one-way ANOVA followed by Dunnett’s test). EA – erucic acid; TAC – total antioxidant capacity; TMZ – temozolomide; TOS – total oxidant status

Changes in both TAC and TOS became significant early, at 24 h, starting with the 20 µg/mL EA concentration. TAC values dropped, only to slightly recuperate by hour 72, whereas the TOS values even slightly worsened by this time (Figure 3).

### Apoptotic and antiapoptotic gene expression

**Figure 4:**
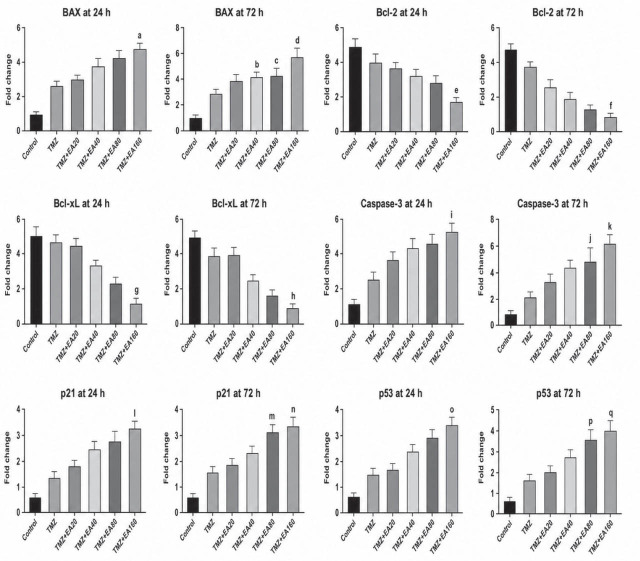
Relative mRNA expression of Bax, Bcl-2, Bcl-xL, caspase-3, p53, and p21 following the treatment of U87 MG cells with TMZ (200 ng/mL) alone or in combination with EA (20, 40, 80, and 160 µg/mL) for 24 h and 72 h. The data are presented as means ± SD of 3 independent biological replicates. Within each replicate, five technical measurements were averaged to obtain one value. ^a^P=0.032; ^b^P=0.028; ^c^P=0.019; ^d^P=0.008; ^e^P=0.008; ^f^P=0.004; ^g^P=0.009; ^h^P=0.006; ^i^P=0.032; ^j^P=0.006; ^k^P=0.002; ^l^P=0.032; ^m^P=0.006; ^n^P=0.002; ^o^P=0.028; ^p^P=0.019; ^q^P=0.004 compared to control (one-way ANOVA followed by Dunnett’s test). EA – erucic acid; TMZ – temozolomide

The expression of apoptosis-related genes confirms the enhanced cytotoxicity of TMZ by EA evidenced above (Figure 4). Compared to control at 24 h, Bax mRNA expression was significantly greater only in the TMZ+EA160 group. By hour 72, it also became significant in the TMZ+EA40 and TMZ+EA80 groups, while it increased even further in the TMZ+EA160 group, suggesting a concentration- and time-dependent effect.

A similar concentration and time-dependent trend is evidenced by caspase-3 mRNA expression following TMZ+EA exposure. At 24 h, it was significantly higher in the TMZ+EA160 group, while at 72 h, the difference from control became significant in the TMZ+EA80 and TMZ+EA160 groups, which is consistent with increased apoptosis-associated transcriptional response at later time points involving the p53/p21 axis. At 24 h, p53 mRNA expression increased significantly in the TMZ+EA160 group and by hour 72, it became significantly greater in both the TMZ+EA80 and TMZ+EA160 groups. Similarly, p21 expression increased significantly at 24 h in the TMZ+EA160 group compared to the control and further increased at 72 h in the TMZ+EA80 and TMZ+EA160 groups.

Conversely, the expression of the antiapoptotic genes *Bcl-2* and *Bcl-xL* dropped compared to control. At 24 h, both Bcl-2 and BclxL mRNA levels were significantly lower in the TMZ+EA160 group. This decrease became even more pronounced at 72 h.

Overall, the coordinated upregulation of Bax, caspase-3, p53, and p21 and downregulation of antiapoptotic Bcl-2 family members evidence a transcriptional profile consistent with the activation of intrinsic apoptotic pathways, particularly at high EA concentrations and longer exposure durations.

## DISCUSSION

Our results confirm the hypothesis that EA enhances the cytotoxic effects of TMZ in all investigated aspects, including cell viability, oxidative balance, and apoptosis-related gene expression in U87 MG cells. However, these are only preliminary observations providing exploratory evidence that highlights biological tendencies and targetable pathways rather than conclusive indicators directly predictive of clinical outcomes. They therefore warrant further investigation of EA as a potential modulating factor in TMZ-based experimental settings.

The decrease in cell viability and concomitant increase in LDH release in the combination groups suggests substantial cellular injury and compromised membrane integrity (15), which has also been confirmed by progressive morphological deterioration, including rounding, loss of cellular extensions, and shrinkage, especially at 80 and 160 µg/mL EA concentrations. However, as increased LDH levels are typically detected during late-stage apoptosis, our findings may also indicate secondary necrosis or mixed cell death processes (16, 17). Since we did not directly assess specific biochemical markers distinguishing apoptosis from necrotic pathways (such as Annexin V/PI flow cytometry or caspase activity assays), we cannot precisely define the mode of cell death (10, 18).

Furthermore, because our study was not specifically designed to calculate a formal combination index, it is not possible to conclude whether the interaction between TMZ and EA was synergistic or additive.

The increase in TOS and decrease in TAC measured in cell lysates are consistent with the reduction in cell viability and suggest that oxidative imbalance contributed to the cellular response induced by TMZ + EA treatment (19, 20). This interpretation is supported by previous studies showing that TMZ-related cytotoxicity is closely associated with DNA damage and apoptotic pathways (9, 10, 21). In addition, recent studies have reported that EA may promote oxidative stress- or ferroptosis-related cancer cell death in combination with chemotherapeutic agents (4, 5). However, our findings should be interpreted as indicative of a general redox imbalance, as specific ROS subtypes, lipid peroxidation products, and ferroptosis markers were not directly measured.

The gene expression findings are also compatible with apoptotic response linked to this redox imbalance. Increased Bax expression, together with decreased Bcl-2 and Bcl-xL levels, reflects a shift in the balance of Bcl-2 family proteins toward an apoptotic state, in line with greater mitochondrial outer membrane permeability and activation of intrinsic apoptotic pathways (22,23,24). Bcl-xL is associated with chemoresistance in various cancer types, and suppression of this protein has been reported to increase sensitivity to cytotoxic therapies (25). The increase in caspase-3 gene expression is a complementary finding, suggesting the engagement of apoptosis-associated transcriptional responses (16, 23, 26, 27), but caspase activity was not directly quantified, and our interpretations are limited to the mRNA level. Furthermore, the upregulation along the p53/p21 axis suggests that the combination treatment affects not only mitochondrial signalling but also nuclear stress responses and cell cycle regulation (21, 28). p53 is a central regulator at the interface between DNA damage and oxidative stress, whereas p21 is a key cyclin-dependent kinase inhibitor that mediates cell cycle arrest, particularly at the G1/S checkpoint. Their concomitant increase in our study is compatible with a scenario in which cells exposed to damage exceeding their repair capacity tend to limit proliferation and shift toward apoptosis. On the other hand, depending on its binding partners and the tumour context, p21 can sometimes have cytoprotective or antiapoptotic effects (28). For this reason, interpreting p21 elevation alone as a definitive indicator of apoptosis would be inappropriate. A more realistic approach is to contextualise our findings within an integrated framework that considers p21 upregulation together with the Bax/Bcl-2 balance and the increase in caspase-3 (16, 23).

The evaluation of EA, a lipid-derived molecule, in combination with TMZ offers a distinct contribution to the literature that has predominantly been focused on phenolic or antioxidant-type compounds. Previous studies investigating combinations of TMZ with natural or redox-active compounds have described additional cytotoxic effects mediated by enhanced oxidative stress and the potentiation of apoptosis (22,23,24). Yet, like many of these studies, ours is based on an early-stage *in vitro* model using a single cell line. Considering that monolayer cultures like U87 MG fail to fully capture the tumour microenvironment, genetic and phenotypic heterogeneity, and blood-brain barrier characteristics, our findings should be viewed primarily as a hypothesis-generating platform that requires validation in more advanced experimental settings rather than as evidence directly translatable to clinical application (8, 29).

Looking at a broader toxicological context, studies published in *Archives of Industrial Hygiene and Toxicology* have highlighted the relevance of oxidative stress-associated cytotoxicity endpoints in diverse cellular models, emphasising the combined evaluation of viability assays and oxidative stress biomarkers in mechanistic *in vitro* research (30,31,32). Although these investigations address different exposure paradigms, they collectively reinforce the concept that redox imbalance represents a central component of cellular injury responses. Viewed from this perspective, our findings provide additional exploratory evidence regarding redox modulation in glioblastoma cells under combined chemotherapeutic exposure.

### Study limitations and future directions

The findings of this study are based on exploratory data obtained exclusively from the U87 MG cell line under two-dimensional (2D) *in vitro* conditions. Therefore, the results should not be regarded as generalisable evidence that fully captures the biological heterogeneity of glioblastoma but rather as an initial framework that should be interpreted cautiously and refined in subsequent studies.

In addition, our assessments did not include specific ROS species and caspase activity. Future studies incorporating additional cell lines and, preferably, 3D/organoid models supported by Western blot analyses and functional assays, as well as pharmacokinetic and toxicological evaluations in appropriate animal models, will be essential to strengthen the mechanistic and translational value of the present findings.

Another limitation is the absence of an EA-only treatment group. Therefore, future studies should include EA alone at comparable concentrations to distinguish its independent effects from its modulatory role in TMZ-based treatments.

Future investigations should also incorporate direct discrimination between different cell death modalities, including distinct forms of apoptosis and regulated necrosis pathways, as emerging evidence indicates substantial overlap and crosstalk among these mechanisms.

## CONCLUSION

Despite its obvious limitations, our study has clearly indicated that erucic acid increases the effect of temozolomide in terms of lower cell viability, membrane disruption, and shift toward oxidative stress in U87 MG cells, further supported by the observed changes in the expression of p53, p21, Bax, Bcl-2, Bcl-xL, and caspase-3, consistent with a response indicative of apoptosis, which needs to be further investigated with direct cell death phenotyping to draw definitive conclusions.

Overall, our findings should be considered preliminary and may inform future experimental investigations of TMZ-based combinations in additional glioblastoma cell lines, resistance models, 3D culture systems, and appropriate *in vivo* studies.

## Abbreviations

DMEM: Dulbecco’s Modified Eagle Medium
DMSO: dimethyl sulphoxide
EA: erucic acid
FBS: foetal bovine serum
MTT: 3-(4,5-dimethylthiazol-2-yl)-2,5-diphenyltetrazolium bromide
ROS: reactive oxygen species
TAC: total antioxidant capacity
TMZ: temozolomide
TOS: total oxidant status
